# Simulated kangaroo care in very preterm infants does not reduce physiological instability: the COSYBABY randomised controlled cross-over trial

**DOI:** 10.3389/fped.2025.1532848

**Published:** 2025-04-30

**Authors:** Caroline Hartley, Tricia Adjei, Mohammad Chehrazi, Joan Baticula, Izabela Andrzejewska, Matthew Hyde, Neena Modi, Suzan Jeffries

**Affiliations:** ^1^ Department of Paediatrics, University of Oxford, Oxford, United Kingdom; ^2^ Section of Neonatal Medicine, Faculty of Medicine, Chelsea and Westminster Hospital Campus, School of Public Health, Imperial College London, London, United Kingdom

**Keywords:** apnoea, preterm, newborn, desaturation, bradycardia, kangaroo care

## Abstract

**Introduction:**

Infants who are born very preterm experience frequent episodes of physiological instability including apnoea, oxygen desaturation and bradycardia due to immaturity of the pulmonary and nervous systems. Parental contact, such as kangaroo care, may reduce physiological instability. However, there may be long periods when parents cannot be with their baby. The BABYBE SYSTEM® is a medical device designed to simulate kangaroo care.

**Methods:**

We conducted a randomised cross-over trial to determine whether episodes of apnoea and other episodes of physiological instability were reduced when infants were on an active BABYBE mattress. Each infant was included in the study for five consecutive days, with successive 12-h periods of the BABYBE® mattress being switched on or off. Episodes of physiological instability were identified from recordings of the vital signs monitors and compared with clinical notes. Generalised estimating equations models were used to compare physiological instability when the BABYBE mattress was switched on vs. off.

**Results:**

A total of 23 infants born before 32 weeks' gestation were included in the main analysis. There was no significant difference between the number of apnoeic episodes infants experienced in the 12-h period when the BABYBE mattress was on compared with when the mattress was switched off (difference between conditions = 1.5 apnoeas, 95% CI: −0.2–3.2, *p* = 0.09). The number of episodes of apnoea identified from vital signs recordings were much higher than those documented in the clinical records (a total of 1,157 apnoeic episodes were identified across all infants from vital signs recordings compared with a total of 27 documented in clinical/nursing notes of the same infants).

**Discussion:**

This trial does not provide evidence of a benefit of the BABYBE mattress for improving physiological stability in preterm infants. This study provides confirmation of the under-recognition of apnoeic episodes in clinical notes and the benefit of assessing electronic recordings of vital signs to gain a more complete picture of physiological stability.

## 1 Introduction

Infants born very preterm spend their first few months of life in a neonatal unit and can be separated from their parents for long periods. This may have a negative impact on the infants, including on cardiorespiratory regulation. Preterm infants are physiologically unstable due to the immaturity of their pulmonary and nervous systems (1–3). Apnoea of prematurity (AOP) is common, occurring in more than 85% of infants born below 31 weeks' gestation (4, 5), and this may in fact be an underestimate due to inaccuracies of the measurement of apnoea on hospital monitors (6–8). Episodes of apnoea can lead to substantial drops in oxygen saturation and heart rate, and may result in poorer neurodevelopmental outcomes and increased risk of retinopathy of prematurity (1, 9).

Parental interventions such as skin-to-skin contact, kangaroo care and hearing the mother's voice may have multiple benefits for the infant including reduced risk of mortality, increased weight gain, increased bonding and enhanced cognitive development (10–12). These interventions may also improve physiological stability in infants—reducing episodes of apnoea, oxygen desaturations and bradycardia; reducing heart rate variability; and increasing cerebral blood flow (13–16). Unfortunately, it is not possible for parents to be with their babies all the time, and factors such as childcare for older siblings or long distances between home and the hospital may reduce parental contact. Devices that can mimic aspects of parental presence have been developed with the aim of reducing physiological instability in infants, reducing parental stress and improving bonding. The BABYBE SYSTEM® (Natus Medical Incorporated, United States) is a clinically tested soft robotic medical device designed to simulate kangaroo care whilst the baby is in their incubator or cot. It incorporates a “mother module”, which can detect the mother's heart rate and breathing rate, and a soft gel mattress for the baby to lie on. The mattress has pneumatic pumps that move in accordance with the mother's breathing rate (either in real time or following a recording), and it is heated to skin temperature. Recordings of the mother's voice or music can also be played to the infant.

We hypothesised that when infants were lying on the BABYBE mattress they would experience fewer episodes of physiological instability. Our primary objective was to determine whether the BABYBE mattress reduced episodes of apnoea. We conducted a randomised cross-over trial to investigate whether episodes of apnoea, oxygen desaturation and bradycardia were reduced when infants were placed on an active BABYBE mattress.

## 2 Methods

### 2.1 Study design and participants

This was a single centre randomised controlled cross-over trial conducted at the High Dependency Unit, Neonatal Unit, Chelsea and Westminster Hospital NHS Foundation Trust, London, United Kingdom. Infants were eligible for inclusion in the trial if they were born before 32 weeks' gestational age and likely to remain in the Chelsea and Westminster Neonatal Unit for at least ten days. Infants were excluded if they had major congenital malformations, major brain injury or any condition that the attending consultant considered incompatible with survival. No infants were mechanically ventilated at the time of the study. Written informed parental consent was obtained for all infants. Approval was obtained from the National Research Ethics Service (reference 20/LO/0205). The study sponsor was Chelsea and Westminster NHS Foundation Trust. The trial conformed to the standards set by the Declaration of Helsinki and Good Clinical Practice.

Each infant was included in the study for five consecutive days. Each day was divided into two 12-h periods (08.00–20.00; 20.00–08.00). Infants were studied with consecutive 12-h periods of the BABYBE mattress being switched on or off (with no washout period). Whilst in the initial protocol we planned for the infants to be off the BABYBE mattress completely (and receiving the standard care mattress) during the off periods, logistically this was not possible as the mattress takes several hours to warm up and can only be kept at the correct temperature when placed in an incubator. Thus, instead during the “off” periods the mattress was paused so that no heartbeat sound or breathing motion were transmitted from the BABYBE mattress. This protocol change was implemented before any infants were studied.

Once consented, each infant was randomly allocated 1:1:1:1 via a computer-generated algorithm to one of four time/intervention sequence: ABABABABAB 8:00 am start; BABABABABA 8:00 am start; ABABABABAB 8:00 pm start; BABABABABA 8:00 pm start, where A is when the mattress is switched off and B is when the infant is lying on the BABYBE mattress whilst switched on. These sequences were combined into two groups for analysis with those infants in the Start-Off sequence (i.e., started with A) combined and those in the Start-On sequence (i.e., started with B) combined. The sequences were combined due to small numbers in each sequence in the final sample. However, circadian rhythms effect physiology (17) and may have affected the results. For completeness, Supplementary Tables 1, 2 provide the results with the four sequences separated for the primary outcome (number of apnoeas).

### 2.2 Trial intervention: the BABYBE®

The BABYBE® mattress (BABYBE SYSTEM®, Natus Medical Incorporated, United States) is a class 1 soft-robotics medical innovation designed to replicate kangaroo care. Pneumatic pumps inflate to replicate movement from the mother's chest due to breathing and the mattress is heated to skin temperature. A sound probe replicates the mother's heartbeat and can be used to relay the mother's heartbeat in real time or play a recording. Speakers can be used to play a recording of the mother's voice or music, though these speakers were not used in our study.

A member of the research team (SJ) oversaw all changes between the BABYBE mattress being on or off (paused), provided assistance to the baby's nurse and ensured that the intervention sequence was undertaken as assigned to each baby. At the end of the five-day trial period, infants returned to receiving the standard care mattress.

Clinical care and parental contact (including the infant being removed from their incubator/cot for feeding and skin-to-skin contact) continued as normal throughout the trial. Skin-to-skin contact was not withheld at any point. Prolonged periods during which the infant was being held by their parents were recorded. The unit has implemented the UNICEF UK Baby Friendly Initiative and allows parents to visit their babies at any time of day or night (24/7). The unit encourages skin-to-skin contact for babies of any gestation, with no limit on the duration for which skin-to-skin can be performed.

### 2.3 Outcome measures

The primary outcome measure was the number of episodes of apnoea during each 12-h study period. An episode of apnoea was defined as cessation of breathing that lasts for more than 15 s (18). Secondary outcome measures were the number of episodes of oxygen desaturation (defined as less than 80% for at least 10 s) and the number of episodes of bradycardia (defined as less than 100 beats per min for at least 15 s) (19). Note that the target range for oxygen saturation in the unit (for infants receiving oxygen) is 91%–95%. Parent views on the research study and the BABYBE were also investigated as secondary outcomes and have been reported in detail elsewhere (20).

### 2.4 Data collection

The trial was split into two phases according to the method of data collection. There were no differences between Phase 1 and 2 in terms of study design or inclusion/exclusion criteria. In Phase 1, episodes of physiological instability (apnoea, bradycardia, oxygen desaturation) were identified from nursing charts only (note the nurse was not blinded to the intervention). The events are recorded in nursing notes as part of routine care. In Phase 2, episodes of physiological instability were identified from high frequency recordings of the vital signs monitors. For comparison, episodes of physiological instability were also recorded from nursing charts for infants recruited during Phase 2. As the vital signs recordings provide a much higher level of sensitivity for detecting events (8), the primary statistical analysis was conducted using data collected during Phase 2. The trial was split into the two phases as during the course of the trial the algorithm to detect apnoeas from electronic recordings was finalised and it became clear that using electronic recordings provides much greater detail with regard to apnoeas compared with clinical notes (8).

Infants' vital signs in Phase 2 were recorded continuously using an IntelliVue MX700 Phillips monitor and downloaded using VitalRecorder software (VitalDB) (21) to a recording laptop connected to the infant's monitor. Heart rate and oxygen saturation were monitored using ECG and a pulse oximeter respectively and recorded at a sampling rate of 1 Hz. ECG was recorded at a sampling rate of 500 Hz and the impedance pneumograph (IP) at a sampling rate of 62.5 Hz.

### 2.5 Vital signs recording analysis

The two investigators (TA and CH) who analysed vital signs recordings (identifying episodes of apnoea and other physiological events) were blinded to intervention sequences. Note that CH and TA were not involved in study design or data collection. Analysis was conducted in MATLAB R2020a (Mathworks, USA). To identify episodes of apnoea, inter-breath intervals (IBI) were first identified from the IP using the algorithm described by Adjei et al. (8), which was developed and validated for the identification of IBI and apnoeas in preterm infants. The algorithm uses the IP signal to detect individual breaths made by the infant, and consequently also apnoeas. Briefly, the algorithm uses an adaptive amplitude threshold to identify the timing of individual breaths within the IP signal. Prior to thresholding, cardiac artefact is removed from the IP signal using the ECG signal (7, 8); during periods where the ECG signal was not recorded (due to technical problems), cardiac artefact was not removed from the IP signal and a higher amplitude threshold was used (8) (ECG was missing in 7 infants with a range of 65%–100% of the signal missing, in all other infants the ECG was recorded throughout). The IP signal was high-pass filtered at 0.5 Hz to remove low frequency movement artefact, and periods of gross movement artefact which resulted in the signal reaching the hard-limited upper or lower values were removed from the analysis. Finally, an automated classifier trained [in the independent sample of infants studied by Adjei et al. (8)] to differentiate periods of true apnoea from artefactually low amplitude signal was applied to all episodes with an IBI greater than 15 s.

All episodes identified as a true apnoea from this algorithm were visually checked by one of the investigators to determine that the IP signal during this period was free from prolonged gross movement artefact or shallow breathing (investigators were blinded to whether the mattress was on or off). A total of 257 out of 2,118 (12%) of the episodes initially identified by the classifier were removed following visual inspection [this is similar performance to the test set used in the development of the algorithm which had a false positive rate for the identification of apnoeic episodes of 14% (8)]. Only those episodes identified as a true apnoea by the classifier and verified by visual inspection were included in the analysis.

The number of episodes of apnoea, oxygen desaturation and bradycardia were assessed within 12-h recording windows. Episodes of oxygen desaturation were identified from the monitor-derived measure of oxygen saturation and episodes of bradycardia from the monitor-derived measurement of the heart rate from the ECG signal. Where the ECG signal was not available due to technical difficulties the pulse from the pulse oximeter was used instead. Episodes of apnoea, oxygen desaturation or bradycardia that occurred within 60 s of the previous episode were counted as single events. Periods of interrupted signal, indicative of movement artefacts or loss of signal due to the displacement of the electrodes or probes, were removed from the analysis (8). It is not possible to impute this data. On average 10.6% of each 12-h recording period was missing (median: 1.3 h out of each 12-h period, interquartile range: 0.6–1.9 h). The amount of missing signal was not significantly different when the BABYBE mattress was switched on compared to the 12-h periods when the mattress was off (*p* = 0.71).

### 2.6 Statistical analysis

Stata version 14 was used for all statistical analyses (Stata Corp., College station, TX, United States). A power calculation determined that 56 infants would provide 80% power to detect a 10% difference in the frequency of apnoea when the infant is on the BABYBE mattress compared to when the mattress was switched off. Statistical analysis was conducted as intention-to-treat. Where data from a whole 12-h period was missing (for example, due to technical difficulties or missing recordings), data were estimated using multiple imputation methods [for the primary outcome measure—number of apnoeas—data were missing for 12 out of a total of 276 (4%) recording periods across all infants]. One infant with missing recordings throughout the entire five-day recording was removed from the analysis (i.e., data were not imputed for this infant). To fully describe the distributions, data are presented as mean and standard deviation, and median, interquartile range and range. Generalised estimating equations (GEE) models were used to estimate the differences between the two study arms while accounting for within-subject correlations arising from the crossover design. Analysis of variance (ANOVA) models were used to estimate period, sequence, and carryover effects. Mean differences and confidence intervals are presented with the BABYBE mattress off as the reference condition. Bias-corrected confidence intervals were calculated via a bootstrap method with 1,000 iterations due to the small sample size.

## 3 Results

**Figure 1:**
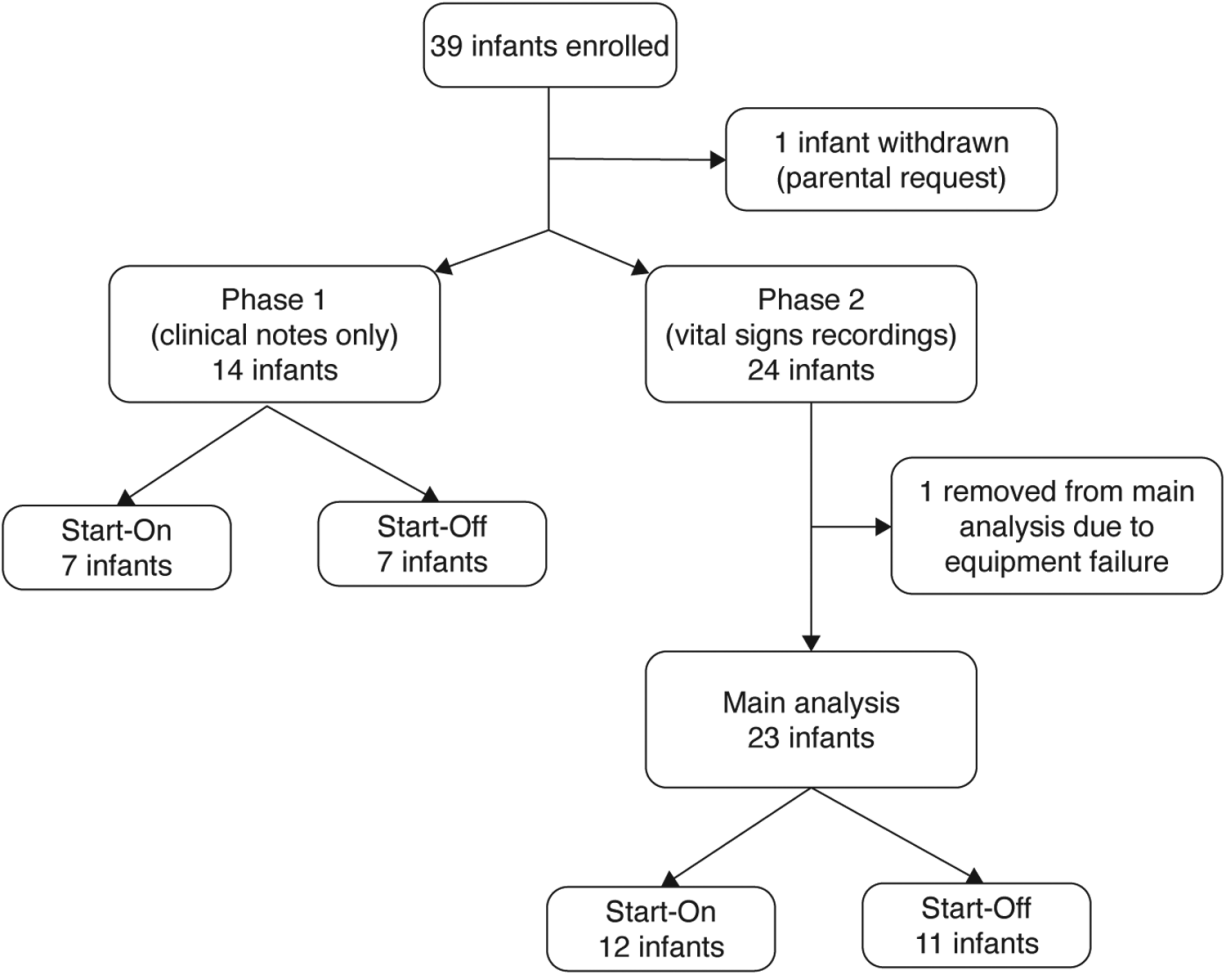
Participant flow chart.

The study was conducted from January 2020 until March 2022. The study was stopped as the manufacturer made a business-related decision to cease production of the BABYBE® mattress. At the same time, study funding ended and given the cessation of production of the mattress, it was not possible to apply for further funding. A total of 39 infants were recruited before the study was stopped (Figure 1). The first 14 infants were studied during Phase 1 where episodes of physiological instability were documented from nursing records only (Supplementary Results, Supplementary Figure 1). A total of 23 infants were recorded during Phase 2 and contributed to the main statistical analysis (Figure 1). Infant demographic information is given in Table 1. All of the results presented in the main text are for infants studied during Phase 2, see the Supplementary Material for the results from Phase 1.

**Table 1:** Infant demographic information.

Demograhpic characteristic	All infants	Start OFF	Start ON
Sex			
Boys	13 (56.5%)	6 (54.6%)	7 (58.3%)
Girls	10 (43.5%)	5 (45.5%)	5 (41.7%)
Mode of delivery			
VD	9 (39.1%)	4 (36.4%)	5 (41.7%)
ILCS	2 (8.7%)	0 (0%)	2 (16.7%)
PLCS	12 (52.2%)	7 (63.6%)	5 (41.7%)
Birth weight (g, mean ± SD)	994.1 ± 339.7	980.5 ± 252.9	1,006.6 ± 415.1
Weight at study commencement (g, mean ± SD)	1,441.7 ± 429.0	1,267.8 ± 339.7	1,601.1 ± 453.0
Gestational age (weeks)	27.6 ± 2.5	28.1 ± 1.9	27.2 ± 2.9
Postmenstrual age (weeks)	32.7 ± 2.4	32.2 ± 2.6	33.1 ± 2.2
Maternal age (years)	34.3 ± 4.2	34.8 ± 5.3	33.9 ± 3.1

For infants included in the main statistical analysis (*n* = 23) with vital signs recorded. Demographic characteristics are shown overall and by two study groups starting either with the BABYBE ON or OFF. Data are shown as number of infants (%) or mean ± standard deviation. VD, vaginal delivery; ILCS, in-labour Caesarean section; PLCS, pre-labour Caesarean section; SD, standard deviation.

### 3.1 Episodes of physiological instability in vital signs recordings were much higher than documented in clinical notes

**Figure 2:**
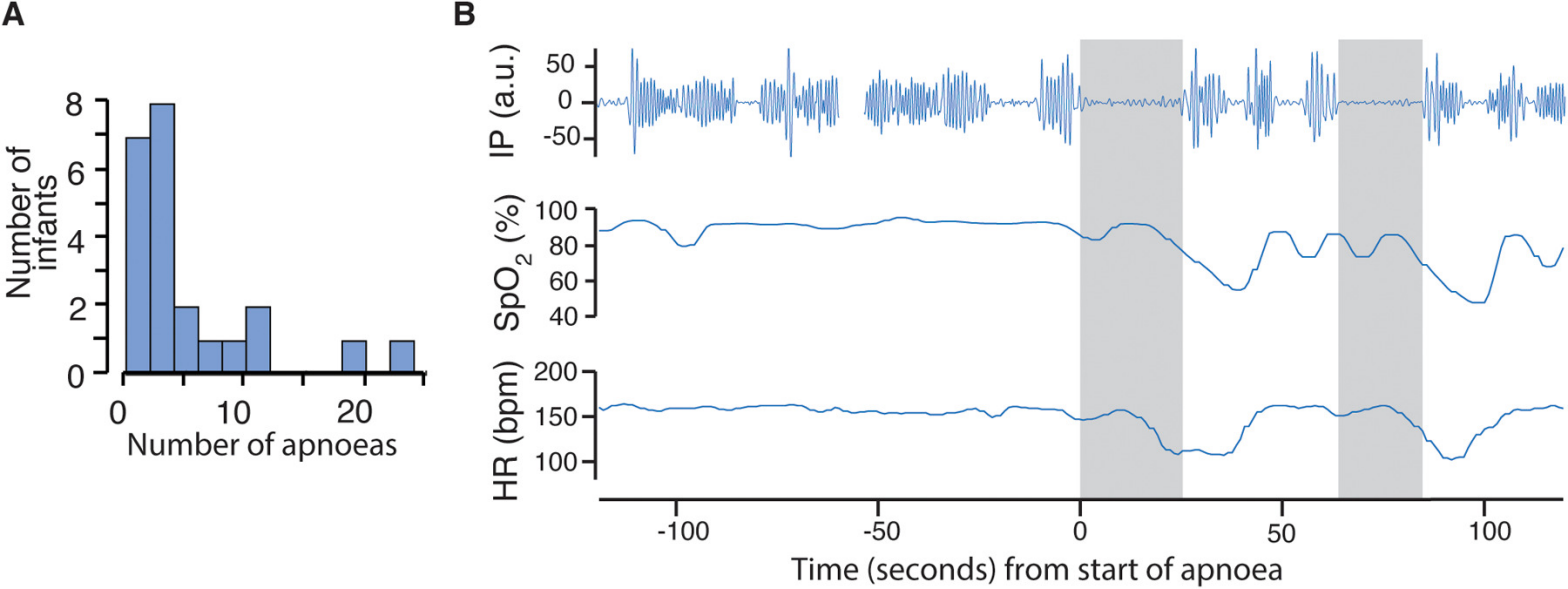
Frequency of episodes of apnoea and example recording. **(A)** Histogram of number of apnoeas in each infant averaged over the 12-h recording periods (including periods when the BABYBE mattress was on and off). **(B)** An example apnoea identified from the vital signs recordings. Top row shows the impedance pneumography (IP) signal, with clear oscillations indicating chest wall movement before the apnoea. An apnoea of 23 s is indicated by the grey shaded area, detected using the algorithm developed and validated in independent infants (8) (see Methods). The heart rate and oxygen saturation subsequently drop. A second apnoea of 20 s also causes a drop in heart rate and oxygen saturation.

On average infants experienced a median [range] of 2 ([0–79]) episodes of apnoea in a 12-h period (Figure 2A). The number of apnoeas detected from recordings of the vital signs data were much higher than those documented in clinical notes: the total number of apnoeas documented in clinical notes across all 23 infants and recording sessions was 27, compared with a total of 1,157 identified on vital signs recordings. Figure 2B shows an example of an apnoea identified from the vital signs recordings not documented in the clinical notes.

The variability in physiological stability between infants was also apparent for episodes of oxygen desaturation and bradycardia (oxygen desaturation:, median in a 12-h period: 11, range: 0–64, total: 2,791; bradycardia: median: 1, range: 0–12, total: 428). The number of these episodes were higher on the vital signs' recordings compared with those documented in the clinical notes (oxygen desaturation: total number of episodes recorded in clinical notes: 1,249; bradycardia: 313).

### 3.2 The BABYBE® mattress did not have a significant effect on physiological stability

**Figure 3:**
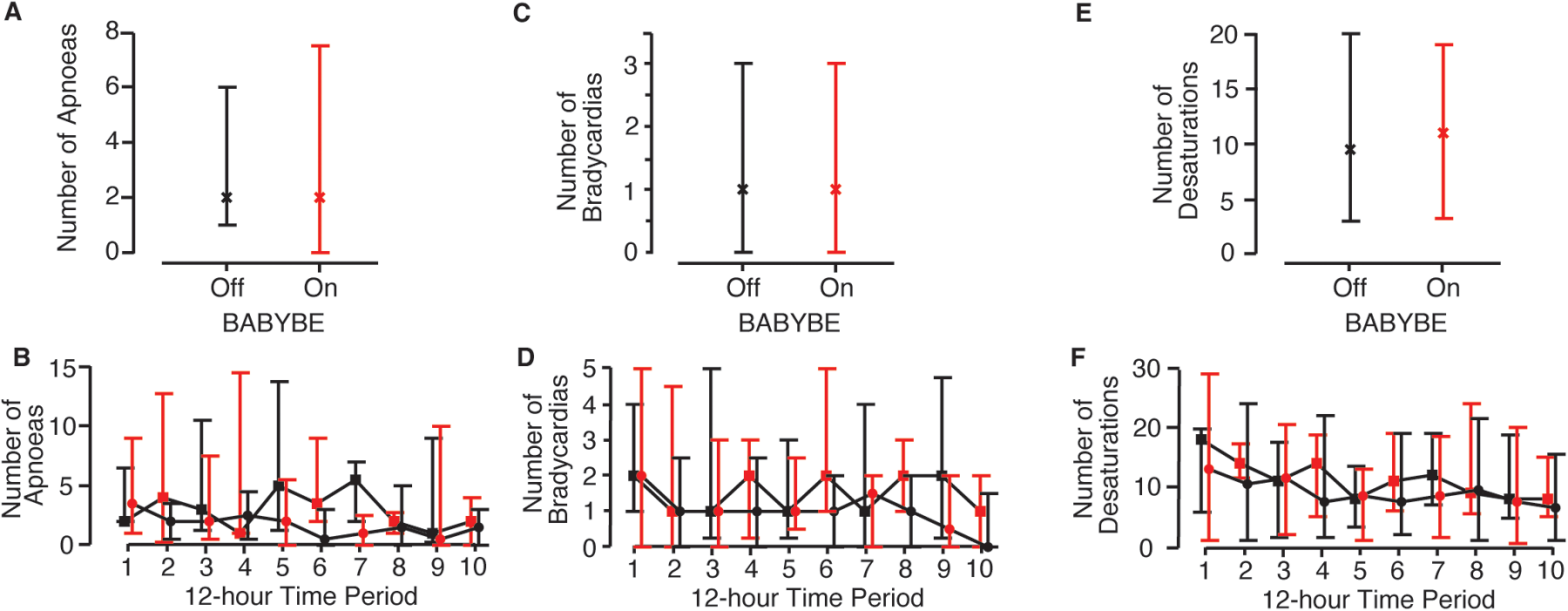
Episodes of physiological stability when infants were on the active BABYBE mattress compared with when the mattress was off. Average number of apnoeic episodes **(A,B)**, bradycardias **(C,D)** and oxygen desaturations **(E,F)** when infants were on the active BABYBE mattress (red) compared with when the mattress was off (black). Data is presented as an average across all 12-h recording periods **(A,C,E)** and across each of the 12-h recording periods **(B,D,F)**. The infants were randomised to initially start the trial on the BABYBE mattress (*n* = 12, indicated with circular points in **B,D,F**) or with the mattress switched off (*n* = 11, indicated with square points in **B,D,F**). Crosses/points indicate the median and error bars indicate the lower and upper quartiles.

The number of apnoeic episodes infants experienced (the primary outcome measure) did not differ significantly when the BABYBE mattress was switched on compared to the 12-h periods when the mattress was off (Figures 3A,B, Table 2, difference between conditions = 1.5, 95% CI: −0.2 to 3.2, *p* = 0.09, GEE model). Individual infant results are shown in Supplementary Figure 2. Similarly, there was no difference in the number of oxygen desaturations or bradycardias experienced by the infants when the BABYBE mattress was on compared to when the mattress was off (Figures 3C–F, Table 2, oxygen desaturations: difference between conditions = −0.04, 95% CI: −1.4 to 1.3, *p* = 0.95; bradycardia: difference = 0.06, 95% CI: −0.3 to 0.4, *p* = 0.74). Infants spent mean [standard deviation] of 0.6 [1.0] h out of their cot (and off the mattress, often in skin-to-skin care with their parents) during the periods when they were on the active BABYBE® mattress and 0.7 [1.0] h during the periods when the mattress was switched off.

**Table 2:** Main results.

Event type	BABYBE ON	BABYBE OFF	*p*-value
Number of apnoeas	2 [7.5], 0–79	2 [5], 0–41	0.09
Number of desaturations	11 [15.8], 0–54	9.5 [17], 0–64	0.95
Number of bradycardias	1 [3], 0–8	1 [3], 0–12	0.74

Number of events per 12-h period when the infants were on the BABYBE mattress compared with when the mattress was switched off. Numbers are median [interquartile range], range. Averages across each of the 12-h recording periods are shown in Figure 3.

## 4 Discussion

Parental contact, for example, through skin-to-skin or kangaroo care, can improve physiological stability in preterm infants. We investigated whether the BABYBE mattress, which is designed to simulate parental kangaroo care through reproducing breathing and heart rate of the mother and by playing voice recordings, can reduce episodes of physiological instability. The trial was stopped early and hence was underpowered to identify an effect. Nevertheless, there was no suggestion of a difference in the number of episodes of apnoea, bradycardia or oxygen desaturation when the infants were on the BABYBE mattress. However, the mattress improved bonding and reduced parental anxiety, the results of which are reported elsewhere (20).

Our results are similar to a previous within-subject study of 20 preterm infants by an independent research group, which found that whilst heartrate variability decreased during and after periods of kangaroo care, this effect was not observed whilst the infant was lying on a BABYBE mattress (22). The authors suggested that this might be because the mother's heartbeat and breathing are not sensed by the baby in the same way through the mattress as with skin-to-skin contact, and other aspects of kangaroo care such as visual and olfactory stimuli are not present when the infant is on the BABYBE mattress (22). Indeed, the benefits of skin-to-skin contact may be related to co-regulation, with interaction between the baby and their carer which cannot be achieved through interventions such as the BABYBE mattress.

A limitation of our study is that, whilst initially we had planned to remove the infant completely from the BABYBE mattress during the off periods, we were not able to do this due to the long time it takes for the mattress to warm up. It would have been beneficial to compare the apnoea rates to a period when infants were on a standard mattress (recording a baseline period for example) as it may be that the BABYBE confers advantages even when switched off. However, we did not record vital signs when the infants were receiving standard care. Moreover, we did not withhold skin-to-skin contact/parental contact during the study—our unit encourages skin-to-skin care as much as possible. We did not want to restrict skin-to-skin care during the study, however, this may have impacted the results. Additionally, it was not possible for the clinical team or the research staff collecting the data to be blinded to the study intervention. Nevertheless, the investigators conducting the analysis of the vital signs were blinded to the intervention sequences of each infant.

A further limitation of our study was the small numbers of infants. Although the BABYBE mattress had no overall effect on physiological stability in this group of infants, it may be that some individual infants might benefit from the mattress. For example, infant's with very low apnoea rates are unlikely to benefit, whereas those with high apnoea rates may benefit more. However, the presence of apnoea was not an inclusion criterion for our trial, and some infants did not experience any apnoea. Other trials that have demonstrated significant effects of medical devices on apnoea rates have only included infants with apnoea (23). Of note, although there was no effect at the group level, some individual infants in our trial did have lower apnoea rates when lying on the active BABYBE mattress (Supplementary Figure 2). Due to the small sample size, we did not investigate this further here, but sub-group analysis to identify particular groups where such technology is beneficial are needed. Finally, we did not record factors such as feeding time, position of the infant, and medication. These could have affected the vital signs, particularly given the recording window of five days. Nevertheless, they would be unlikely to affect all infants systematically in the same way in relation to the timings of the BABYBE and so are unlikely to have had a large impact on our results.

In this trial we used electronic recordings of the infants' vital signs taken directly from the hospital monitors to obtain an accurate picture of the physiological stability of an infant. We also used an algorithm recently developed and validated for the identification of apnoeas in preterm infants (8). Consistent with previous reports (6, 8), the number of episodes of apnoea identified from the electronic recordings was dramatically increased compared with clinical notes (only 2% of apnoeic episodes identified on electronic recordings were documented in clinical notes). The lower numbers of apnoeic episodes recorded on clinical notes is likely due to the known problems of identification of apnoea by the monitors (6–8) and the consequent difficulties for the clinical team to observe these events, particularly when episodes of apnoea are relatively short and self-resolving. As expected, the number of desaturations and bradycardias recorded in clinical notes was somewhat more consistent (than for apnoea) compared with those identified directly from vital signs recordings; however, many events were still missed in clinical notes. Assessing the recordings directly affords for a more accurate assessment of the physiological stability of the infants and was a strength of this study. This study adds to recent work suggesting that measuring physiological stability through electronic recordings is essential to properly assess changes in vital signs (19, 24–27). Moreover, although the majority of episodes detected from electronic recordings are likely to be self-resolving (as they were not recorded in clinical notes), even short pauses in breathing can lead to significant changes in physiology including desaturation and suppression of brain activity and therefore may have a long-term effect on development (28, 29).

In summary, we conducted a randomised controlled cross-over trial to investigate whether episodes of apnoea are reduced when infants are lying on a BABYBE mattress. The study was stopped early, but there was no indication of a difference in physiological stability in infants when they were on the BABYBE mattress compared with periods where the mattress was switched off. By using recordings of vital signs from the infants' monitors, and a recently developed algorithm for the identification of apnoea in preterm infants, we demonstrate that this gives a more detailed and rigorous assessment of an infant's physiological stability compared with clinical notes.

## Data Availability

The datasets presented in this article are not readily available because ethical restrictions apply to the use of this data. Requests to access the datasets should be directed to n.modi@imperial.ac.uk.
