# Supplementary material for: Simulated kangaroo care in very preterm infants does not reduce physiological instability: the COSYBABY randomised controlled cross-over trial
Source: Front Pediatr. 2025 Apr 30;13:1532848. doi: 10.3389/fped.2025.1532848 (PMC12075288; doi:10.3389/fped.2025.1532848)
Supplement: Supplementary file 1 [file Datasheet1.docx]

**Supplementary Material: Physiological stability in very preterm infants during simulated kangaroo care: the COSYBABY randomised controlled cross-over trial**

Caroline Hartley, Tricia Adjei, Mohammad Chehrazi, Joan Baticula, Izabela Andrejewska, Matthew Hyde, Neena Modi, Suzan Jeffries

**Supplementary Results**

The primary statistical analysis was conducted on 23 infants where episodes of physiological instability were identified from recordings of the vital signs monitor. Here we present the results where episodes of physiological instability were identified from the nursing notes. This includes a further 14 infants recruited during Phase 1 and 1 infant recruited during Phase 2 where the monitor data was missing due to technical difficulties. A total of 38 infants were included in this analysis; 19 infants were randomised to the Start-Off sequence and 19 infants to the Start-On sequence. Infant demographics are given in Supplementary Table 3.

Infants experienced a mean [standard deviation] of 0.07 [0.58] episodes of apnoea in a 12-hour period (recorded in clinical notes) whilst they were on the BABYBE mattress compared with 0.09 [0.70] whilst the mattress was off. The median [interquartile range] whilst the infants were on the active BABYBE mattress was 0 [0] and then the mattress was off was also 0 [0]. Infants experienced an median [interquartile range] of 5.5 [7] episodes of oxygen desaturation and 1 [2] episodes of bradycardia (in a 12-hour period, recorded in clinical notes) whilst they were on the BABYBE mattress. Whilst the mattress was off, they experienced an average of 4 [5.25] episodes of oxygen desaturation and 1 [2] episodes of bradycardia (Supplementary Figure 1).

**Supplementary Tables**

**Supplementary Table 1. Apnoea rate for each treatment period and sequence.** Median apnoea rate considering the four sequences which infants were randomly allocated to separately – note that in the main results, the start on and start off sequences are combined due to the small number of participants in each sequence.

|  | **Treatment sequence** | | | |
| --- | --- | --- | --- | --- |
| **Treatment Period** | Start with OFF in morning  **Median (IQR)**  **Sample size: 6** | Start with ON in morning  **Median (IQR)**  **Sample size: 5** | Start with OFF in evening  **Median (IQR)**  **Sample size: 5** | Start with ON in evening  **Median (IQR)**  **Sample size: 7** |
| 1 | 2 (1) | 2 (6.5) | 5 (7.5) | 5 (14) |
| 2 | 6 (12) | 2 (3.5) | 1 (12.75) | 2 (2.5) |
| 3 | 5 (7) | 1 (3.75) | 2 (17) | 4 (9.25) |
| 4 | 3 (12) | 0 (2.25) | 1 (28) | 3 (2.5) |
| 5 | 4 (9) | 0 (0.5) | 5 (14.25) | 5 (6) |
| 6 | 2 (8.25) | 0 (1.5) | 5 (16.25) | 1 (12) |
| 7 | 2 (5.25) | 0 (1) | 7 (6.5) | 2 (13) |
| 8 | 2 (5) | 0 (0.75) | 1 (1.25) | 5 (6.5) |
| 9 | 1 (9) | 0 (4.75) | 4 (11) | 7 (10.25) |
| 10 | 1 (4) | 0 (0.75) | 2.5 (12.5) | 3 (1.5) |

**Supplementary Table 2. Post-hoc statistical analysis for apnoea rate.** The main results combined the infants into two sequences. Here the results are presented with the four sequences separately included.

|  | Partial Sum of square | P value |
| --- | --- | --- |
| Sequence effect | 1104.65 | 0.41 |
| Period effect | 576.82 | 0.14 |
| Carryover effect | 10.78 | 0.61 |
| Treatment effect | 40.96 | 0.32 |
| Total | 17048.89 |  |

**Supplementary Table 3: Infant demographics.** Demographic characteristics are shown overall and by two study groups starting either with ON or OFF BABYBE. VD – vaginal delivery, ILCS – in-labour caesarean section, PLCS – pre-labour caesarean section, SD – standard deviation.

|  | All infants | Start OFF | Start ON |
| --- | --- | --- | --- |
| Sex  Boys  Girls | 18 (46.15%)  21 (53.85%) | 10 (50%)  10 (50%) | 8 (42.11%)  11 (57.89%) |
| Mode of delivery  VD  ILCS  PLCS | 12 (30.77%)  6 (15.38%)  21 (53.85%) | 7 (35%)  4 (20%)  9 (45%) | 5 (26.32%)  2 (10.53%)  12 (63.16%) |
| Birth weight (g, mean ± SD) | 1031.38±353.07 | 1029.50±284.95 | 1033.37±421.26 |
| Weight at study commencement (g, mean ± SD) | 1479.39±481.41 | 1440.32±545.13 | 1518.47±419.53 |
| Gestational age (weeks) | 27.43±2.38 | 27.60±2.01 | 27.26±2.76 |
| Maternal age (years) | 33.64±4.71 | 33.00±4.63 | 34.31±4.82 |

**Supplementary Figures**

**
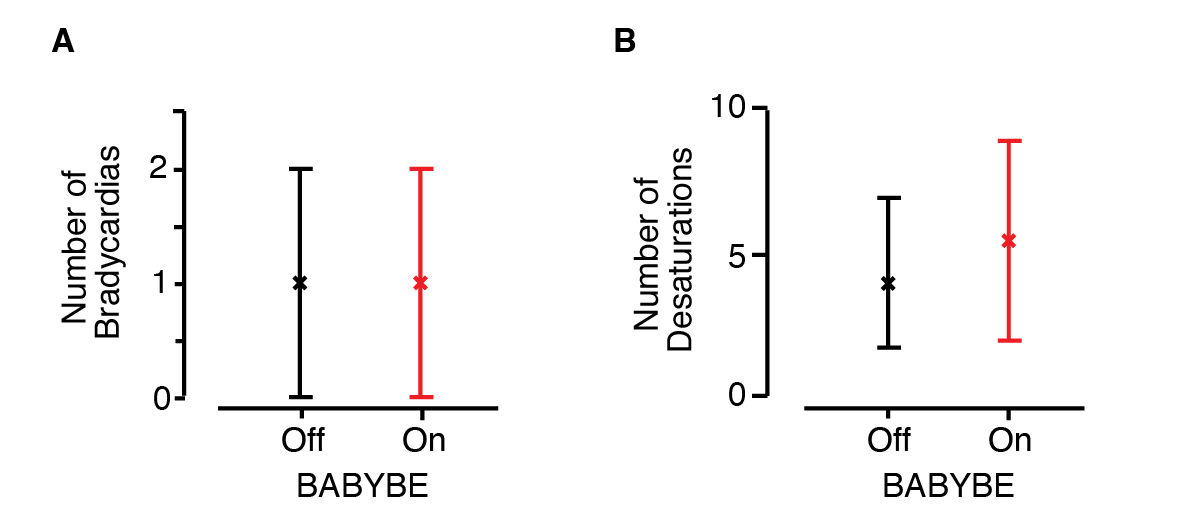
**

**Supplementary Figure 1: Episodes of physiological stability when infants were on the BABYBE mattress compared with when the mattress was switched off, as documented in clinical/nursing notes.** Average number of (A) bradycardias and (B) oxygen desaturations when infants were on the BABYBE^®^ mattress (red) compared with when the mattress was switched off (black). Data is presented as an average across all 12-hour recording periods. All infants (those studied in Phase 1 and Phase 2) are included, n=38. Crosses indicate the median and error bars the lower and upper quartiles. For episodes of apnoea documented on clinical notes, the median, lower quartile and upper quartile were all zero in both groups and so the plot is not presented.

**
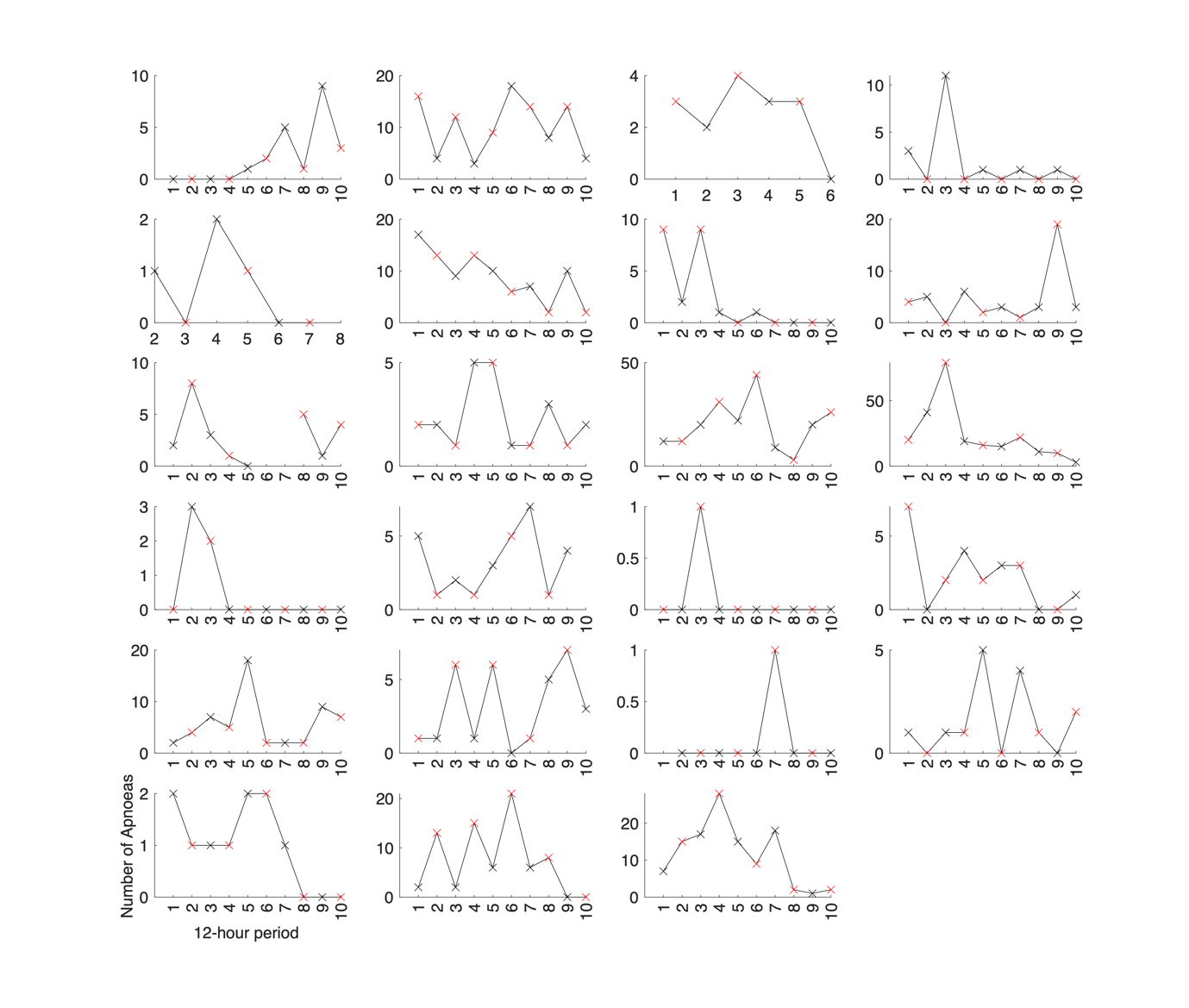
**

**Supplementary Figure 2: Individual infant changes in number of apnoeas across the study period.** Each trace represents an individual infant’s data, showing the 23 infants studied in Phase 2 and indicating the number of apnoeas identified from the vital signs recordings from each sequential 12-hour study period. Black crosses indicate periods when the mattress was switched off and red crosses periods when infants were on the BABYBE mattress (and it was switched on). Infants are shown in order that they were studied. Note each infant is shown on a different y-axis scale according to the number of apnoeas they experienced. Some infants experienced very few apnoeas and so the BABYBE mattress was not likely to confer an advantage. Of interest, some infants exhibit oscillatory patterns.
